# Species diversity and biting activity of *Anopheles dirus* and *Anopheles baimaii* (Diptera: Culicidae) in a malaria prone area of western Thailand

**DOI:** 10.1186/1756-3305-5-211

**Published:** 2012-09-25

**Authors:** Chatchai Tananchai, Rungarun Tisgratog, Waraporn Juntarajumnong, John P Grieco, Sylvie Manguin, Atchariya Prabaripai, Theeraphap Chareonviriyaphap

**Affiliations:** 1 Department of Entomology, Faculty of Agriculture, Kasetsart University, Bangkok, 10900, Thailand; 2 Department of Preventive Medicine and Biometrics, Uniformed Services University of Health Sciences, Bethesda, MD, 20814, USA; 3 Institut de Recherche pour le Développement (IRD), UMR-MD3, Montpellier, France; 4 Department of Mathematics, Statistics and Computer, Faculty of Liberal Arts and Science, Kasetsart University, Kamphaengsean, Nakhonpathom, 73140, Thailand

**Keywords:** *Anopheles dirus*, *Anopheles baimaii*, Exophagic, Zoophilic, Thailand

## Abstract

**Background:**

A survey of adult anopheline mosquito diversities, collected from September 2009 to August 2010, was conducted in a malaria endemic area of western Thailand. Two anopheline species complexes, Dirus and Minimus, along with the Maculatus group were observed. Of several species documented from within each complex and group, four important malaria vectors were identified, including *An. dirus*, *An. baimaii*, *An. minimus,* and *An. sawadwongporni*. Information on biting activity and host preference for any single species within the Dirus complex has never been assessed. Using specific molecular identification assays, the trophic behavior and biting activity of each sibling species within the Dirus complex were observed and analyzed for the Kanchanaburi Province, Thailand.

**Methods:**

Adult female mosquitoes were collected for two consecutive nights each month during a one year period. Three collection methods, human landing indoor (HLI), human landing outdoor (HLO), and cattle baited collections (CBC) were applied. Each team of collectors captured mosquitoes between 1800 and 0600 h.

**Results:**

From a total of 9,824 specimens, 656 belong to the Dirus complex (*An. dirus* 6.09% and *An. baimaii* 0.59%), 8,802 to the Minimus complex (*An. minimus* 4.95% and *An. harrisoni* 84.65%) and 366 to the Maculatus group (*An. maculatus* 2.43% and *An. sawadwongporni* 1.29%). Both *An. dirus* and *An. baimaii* demonstrated exophagic and zoophilic behaviors. Significantly greater numbers of *An. dirus* and *An. baimaii* were collected from cattle as compared to humans (*P* = 0.003 for *An. dirus* and *P* = 0.048 for *An. baimaii*).

**Conclusions:**

Significantly greater numbers of *An. dirus* and *An. baimaii* were collected from cattle baited traps as compared to human landing collections (*P* < 0.05), demonstrating that both species show a strong zoophilic behavior. Knowledge of host-seeking behavior helps to define a species' capacity to acquire and transmit malaria and its contribution to the overall risk for disease transmission in the human population, as well as, assisting in the design and implementation of appropriate vector prevention and control strategies.

## Background

In Thailand, malaria remains one of the most important infectious diseases despite years of well-organized disease control in reducing both mortality and morbidity countrywide [1]. Seventy percent of the malaria cases are documented from the relatively undeveloped borders and hill region of eastern Myanmar, whereas the three species complexes, i.e. *Anopheles dirus*, *An. minimus*, and *An. maculatus* are commonly present and some of them are considered as important malaria vectors, including *An. dirus*, *An. baimaii*, *An. maculatus*, *An. sawadwongporni* and *An. minimus*[2,3].

Better understanding of the behavior of each sibling species within the complex is quite important to help identify their respective roles in disease transmission and to assist the vector control personnel in designing the appropriate steps for vector control management. Due to some complexity within the *An. dirus* complex, the closely related species cannot be differentiated from each other by morphological characters [4-9]. Some species are regarded as excellent malaria vectors because their anthropophagic nature brings them into frequent contact with humans, maintain high parasite loads, and exhibit a highly endophagic behavior [10,11]. Although various studies on the biting patterns and host preference of these complexes have been described in Thailand [12-17], most observations of mosquito biology and behaviors were based exclusively on the populations in which identification was made by morphological characters, except for one study on the *An. minimus* complex recently carried out by Sungvornyothin *et al*. [9]. Such morphological identification of the sibling species within a complex is not reliable and can lead to a high degree of misidentification [9,18]. The application of molecular techniques has made it possible to reliably identify species from entomological surveys. For the *An. dirus* complex, such molecular methods include an allele-specific polymerase chain reaction (AS-PCR), a sequence characterized amplified region (SCAR) and a rapid polymerase chain reaction, which are all considered useful tools for identifying the species within a complex [8,19,20]. In this study, we identified the species within the three complexes in the sympatric area of Pu Teuy Village, Sai Yok District, Kanchanaburi Province. This area is considered to be a malaria prone region with all three complexes responsible for malaria transmission being documented [21,22]. By using a molecular identification assay we were able to observe the trophic behavior, biting activity, and seasonal abundance in each sibling species within the *An. dirus* complex, one of the most important complexes for malaria transmission in Thailand.

## Methods

### Study site

*Anopheles dirus* was collected from the Pu Teuy Village, a village located in Sai Yok District, Kanchanaburi Province, western Thailand (14° 17'N, 99° 11'E). The collection site is located approximately 1 km away from the village. It is surrounded by deep forest lying at approximately 400 m above sea level. Local residents and their occupations are associated with the forest. A narrow, slow running stream (2 m wide and an average of 0.5 m deep) bordered with native vegetation runs across the village. Surrounding vegetation is primarily agricultural land used for growing papaya, cassava and maize and secondarily forest [17].

### Collection methods

Adult female mosquitoes were collected for two consecutive nights each month during a one year period. Three collection methods, including human landing indoor (HLI), human landing outdoor (HLO), and cattle bait collections (CBC) were utilized. Human landing collections were performed in an existing house with all windows closed and one door remaining open throughout the collection period. The entomological team was divided into two groups of four persons each. Two people collected mosquitoes inside the house, whereas the other two people collected outside. The distance between indoor and outdoor collections was set at 100 m. The first team collected from 1800 to 2400 h, followed by the second team that started at midnight and ended at 0600 h. Both teams rotated their starting position between the first and second halves of the evening on each subsequent collection night to avoid collector bias. Collectors also rotated each night between indoor and outdoor locations. Human landing collections occurred uninterrupted for 45 min each hour. Cattle baited collections were conducted by a separate team of two collectors for 15 min each hour [17]. The CBC involved placing a single cow under the untreated cotton bed net, measuring 3.6 m x 3.3 m x 2.0 m (L:W:H) with the net suspended 30 cm above the ground level to allow any hungry mosquitoes to get inside. The same cow was used throughout the study and was placed at least 50 m from the nearest human landing collection site but at equal distances from the forest fringe to avoid a potential distance bias in attracting mosquitoes. All collected mosquitoes were placed in clean, chemical free plastic cups that were labeled by hour and type of collection. Formal human-use approval was granted by the Ethical Research Committee convened by the Research and Development Institute, Kasetsart University, Thailand (KURDI-1/2553-1421457). All collected mosquitoes were held in netting covered plastic cups and were provided with a cotton pad soaked with 10% sugar solution. Each cup was returned to a local processing station in the field for morphological identification. Ambient air temperature and relative humidity were recorded from indoor and outdoor locations and at the site of the cattle baited trap each hour of the collection using a manual thermohygrometer (BARICO GmbH, Villingen-Schwenningen, Germany). Daily rainfall data was also recorded at the study site using a manual Rain Gauge (RAIN GAUGE AUGE KIT, England).

### Morphological and molecular species identification

#### *An. dirus* complex

**Figure 1:**
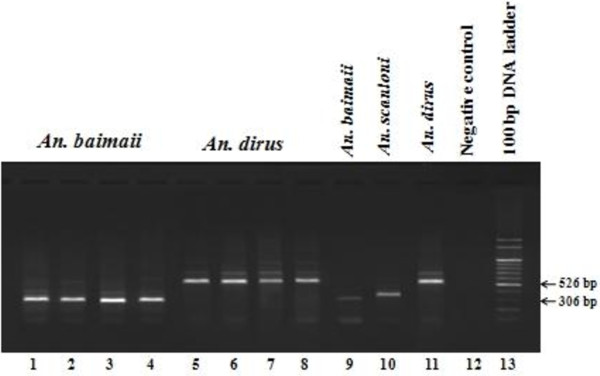
** Multiplex Allele-Specific PCR assay.** Lanes 1–4: *An. baimaii*; lanes 5–8: *An. dirus*; lanes 9–11: positive of *An. baimaii*, *An. scanloni*, *An. dirus*; lane 12: negative control; lane 13: 100 bp molecular ladder.

Members of the *An. dirus* complex were identified to species using illustrated morphological keys for the adult *Anopheles* of Thailand [2,23]. In brief, female mosquitoes with a presector dark spot (PSD) on the radius vein that extended basally beyond the PSD spot on the costa vein and a PSD spot that reached the humeral dark (HD) spot of the costa vein or at least surpassed the middle of the presector pale spot (PSP) of the costa vein of at least one wing were identified as *An. dirus*. Molecular identifications using the AS-PCR assay of Walton *et al*. [8] and Audtho *et al*. [24] were subsequently applied to confirm the species identification, using the specific primers for *Anopheles dirus*, *Anopheles cracens*, *Anopheles scanloni*, *Anopheles baimaii* and *Anopheles nemophilous* (Figure 1). 

#### *An. minimus* complex

Members of the *An. minimus* subgroup were identified to species using illustrated morphological keys for adult *Anopheles* in Thailand [2]. *An. minimus* was identified when a presector pale spot (PSP) was present on the costal vein of both wings, whereas *An. harrisoni* required, at least, the presence of both presector pale and humeral pale spots (HP) on one wing [25]. Molecular identifications were performed using the AS-PCR assay of Garros *et al*. [26], and was conducted using primers specific for *An. minimus* and *An. harrisoni*.

#### *An. maculatus* group

All specimens of the *An. maculatus* group were identified to species using illustrated morphological keys for adult *Anopheles* in Thailand [2]. *An. maculatus* has the R2 + 3 wing veins with one dark spot on both wings and the presector dark (PSD) spot on the R vein is usually shorter than the PSD spots on the subcosta and costa. The R4 + 5 wing veins have 2 dark spots and the abdominal terga segments VII, VIII sometimes on VI are covered with dark scales on the posterolateral corners. Molecular identifications were performed using the AS-PCR assay of Walton *et al*. [27], and were conducted using primers specific for *An. maculatus* and *An. sawadwongporni*.

### DNA extraction

DNA for all *Anopheles* specimens was extracted using Genomic DNA from individual adult mosquitoes, according to the procedures of Linton *et al*. [28] and Manguin *et al*. [20].

### Data analysis

Three key factors were selected for analysis and included seasonality, biting time and collection method. Seasonal periods were identified as wet (June to October), dry (November to February) and hot (March to May), biting times are separated into early evening (1800–2100 hours), late night (2100–2400 h), predawn (2400–0300 h) and dawn (0300–0600 h), and collection types are listed as indoor HLC, outdoor HLC and cattle baited captures.

The nocturnal biting cycle of *An. dirus* and *An. baimaii* were tabulated by averaging the number of *Anopheles* landing per hour per human by indoor and outdoor location and by averaging the number of mosquitoes captured per cow per hour. Comparisons of landing data were analyzed by non-parametric Kruskal-Wallis tests. The accepted level of significance was determined at 0.05% (*P*-valve < 0.05), followed by correlation coefficient (*r*) analysis taking into account the correlation between mosquitoes captured and environmental variables. All data were analyzed using the SPSS statistical package (version 17.0, SPSS, Chicago, IL).

## Results

Observations on adult anopheline diversity, collected from September 2009 to August 2010 at Pu Teuy Village, Kanchanaburi Province, western Thailand are given in Table 1. From a total of 9,824 specimens, 656 belong to the *An. dirus* complex (6.68%), 8,802 were from the *An. minimus* complex (89.6%) and 366 were from the *An. maculatus* group (3.72%). Six important species were molecularly identified to include *An. dirus*, *An. baimaii*, *An. minimus*, *An. harrisoni*, *An. maculatus* and *An. sawadwongporni* (Table 1). Among these, four species, *An. dirus*, *An. baimaii*, *An. minimus* and *An. maculatus* are considered to be important malaria vectors in Thailand [1,29]. In the study, the two most recognized malaria vectors, *An. dirus* and *An. baimaii*, were further characterized to obtain some bionomic information on the biting activity, host preference and density. 

**Table 1:** **Monthly frequency of *Anopheles* mosquitoes at Pu Teuy Village, Sai Yok District, Kanchanaburi Province, for one year (September 2009-August 2010) **

**Month**	***Anopheles*** **complexes**						
	***An. dirus***		***An. minimus***		***An. maculatus***		**Total**
	**DIR**	**BAI**	**MIN**	**HAR**	**MAC**	**SAW**	
September	30	3	9	372	0	0	414
October	54	9	7	234	0	0	304
November	23	4	35	939	0	0	1001
December	0	1	21	676	0	0	698
January	0	0	65	824	0	0	889
February	0	0	64	1115	0	0	1179
March	0	0	34	713	4	0	751
April	4	0	31	554	26	8	623
May	17	4	43	873	43	30	1001
June	51	5	89	783	43	30	1001
July	124	13	74	994	77	42	1324
August	295	19	14	239	50	35	652
Total	598	58	486	8316	239	127	9824

DIR: *An. dirus*, BAI: *An. baimaii*, MIN: *An. minimus*, HAR: *An. harrisoni*, MAC: *An. maculatus*, SAW: *An. sawadwongporni.*

Table 2 provides the monthly distribution of *An. dirus* and *An. baimaii* collected by the three collection methods during the one year study period. From a total of 656 specimens in the *An. dirus* complex, 598 specimens (91.2%) were *An. dirus* and 58 (8.8%) were *An. baimaii*. Greater numbers of these two species were collected during the rainy season (June to October) with a distinct peak in August. For *An. dirus*, 378 (63.2%) were captured on cattle, 168 (28.1%) were collected from outdoor human landing collection, and 52 (8.7%) were obtained from indoor human landing collection. In contrast, a total of 58 specimens of *An. baimaii* were captured from all three collection methods. Twenty-nine (50%) were captured on cattle, 23 (39.7%) were obtained from outdoor human landing collection, and 6 (10.3%) from indoor human landing collection (Table 2). Our results showed that *An. dirus* was more attracted by cattle than humans and, in the latter case, more outdoors than indoors, regardless of the time periods and climatic seasons.

**Table 2:** **Total of monthly captures from three collection methods of *Anopheles dirus* and *Anopheles baimaii* from Pu Teuy Village, Sai Yok District, Kanchanaburi Province **

**Month**	***An. dirus***				***An. baimaii***				**T**	**H**	**R**
	**In(%)**	**Out(%)**	**Cow(%)**	**Total(%)**	**In(%)**	**Out(%)**	**Cow(%)**	**Total(%)**			
Sep	7(23.3)	9(30)	14(46.7)	30	0(0)	0(0)	3(100)	3	28.97	78.38	168.70
Oct	8(14.8)	4(7.4)	42(77.8)	54	1(11.1)	3(33.3)	5(55.6)	9	28.20	79.44	156.90
Nov	0(0)	5(21.7)	18(78.3)	23	1(25)	3(75)	0(0)	4	20.08	85.44	156.90
Dec	0(0)	0(0)	0(0)	0	1(100)	0(0)	0(0)	1	22.47	78.37	7.00
Jan	0(0)	0(0)	0(0)	0	0(0)	0(0)	0(0)	0	20.37	83.47	12.10
Feb	0(0)	0(0)	0(0)	0	0(0)	0(0)	0(0)	0	26.11	77.63	0.00
Mar	0(0)	0(0)	0(0)	0	0(0)	0(0)	0(0)	0	25.63	83.06	76.50
Apr	0(0)	0(0)	4(100)	4	0(0)	0(0)	0(0)	0	27.33	84.01	26.00
May	2(11.8)	3(17.6)	12(70.6)	17	1(25)	2(50)	1(25)	4	26.36	83.11	63.00
Jun	1(1.9)	14(27.5)	36(70.6)	51	0(0)	0(0)	5(100)	5	26.06	92.08	183.00
Jul	12(9.7)	50(40.3)	62(50)	124	1(7.6)	6(46.2)	6(46.2)	13	24.86	88.83	291.00
Aug	22(7.5)	83(28.1)	190(64.4)	295	1(5.2)	9(47.4)	9(47.4)	19	25.80	87.25	208.00
Total	52	168	378	598	6	23	29	58			
(%)	8.7	28.1	63.2		10.3	39.7	50.0				

R: Rainfall (mm), H: Humidity (%), T: Temperature (°C).

**Figure 2:**
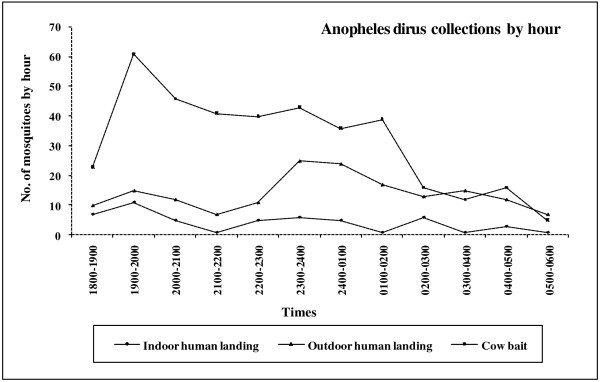
** Temporal patterns of *Anopheles dirus* and blood feeding activity for indoor, outdoor human landing and cattle bait collections.**

**Figure 3:**
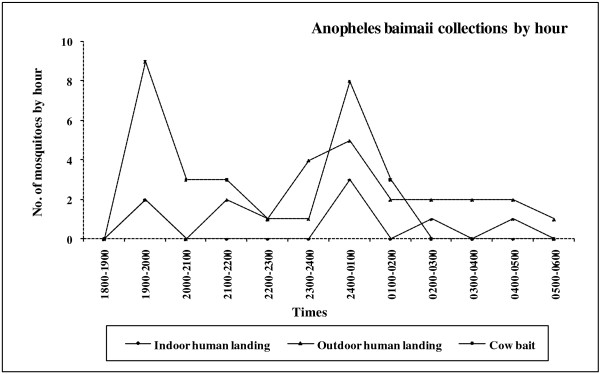
** Temporal patterns of *Anopheles baimaii* blood feeding activity for indoor, outdoor human landing and cattle bait collections.**

Total mosquito biting frequencies by hour and collection method for *An. dirus* and *An. baimaii* are given in Figures 2– 3. *An. dirus* specimens were found to exceed *An. baimaii* in numbers for all collection periods. The indoor biting activity of *An. dirus* presented one prominent peak between 1900 and 2000 h and a smaller peak between 0200 and 0300 h (Figure 2). The outdoor human landing activity was elevated from 2300 to 2400 h (Figure 2). In contrast, cattle baited collections showed one clear peak for *An. dirus* in the early evening (1900–2000 h) followed by a decline throughout the rest of the night (Figure 2). Although low numbers of *An. baimaii* were obtained, the outdoor activity peaks were clearly defined with a distinct outdoor peak between 2400 and 0100 h whereas the indoor peak was difficult to determine due to the low number of specimens collected (Figure 3). Cattle baited catches showed two clear peaks for *An. baimaii* in the early evening (1900–2000 h) and midnight (2400–0100 h) (Figure 3).

**Figure 4:**
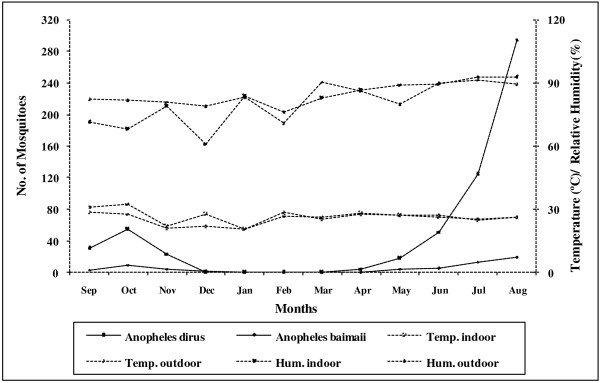
** Monthly collections of *Anopheles dirus* and *Anopheles baimaii* in relation to average ambient air temperature and humidity in Pu Teuy Village, Sai Yok District, Kanchanaburi Province.**

The total number of landing mosquitoes per hour was subjected to a Kruskal-Wallis test, with seasons (dry, hot, and wet), collection methods (indoor and outdoor human bait and cattle bait) and time intervals (early evening, late evening, predawn, and dawn) as discriminating factors. A strong significant difference in the number of *An. dirus* and *An. baimaii* were found between seasons (*χ*^2^ = 70.55; df = 2; *P* < 0.0001 *An. dirus* and *χ*^2^ = 27.34; df = 2; *P* < 0.0001 *An. baimaii*), and between indoor, outdoor human landing and cattle bait (*χ*^2^ = 11.59; df = 2; *P* = 0.003 *An. dirus* and *χ*^2^ = 6.07; df = 2; *P* = 0.048 *An. baimaii*). There was no significant difference in the number of either species collected between the four quarterly evening time intervals (*χ*^2^ = 2.21; df = 3; *P* = 0.529 *An. dirus* and *χ*^2^ = 3.68; df = 3; *P* = 0.298 *An. baimaii*). Data from all collection methods was pooled to determine the correlation between mosquito abundance and environmental variables (Figure 4). *An. dirus* and *An. baimaii* densities strongly correlated with the total rainfall *(r* = 0.454; *P* = 0.016 *An. dirus* and *r* = 0.609; *P =* 0.003 *An. baimaii*) but were not related with relative humidity and minimum or maximum ambient air temperatures *(P >* 0.05).

## Discussion

Of 73 known *Anopheles* species found in Thailand, some members of *An. leucosphyrus* and *An. maculatus* groups and *An. minimus* subgroups, are recognized as important malaria vectors [2]. Five species have been incriminated as malaria vectors in Thailand, including *An. dirus*[15], *An. baimaii*[30], *An. minimus*[31], *An. pseudowillmori*[30] and *An. aconitus*[30,32,33]. In this study, the six species found belong to two groups and one subgroup, including two members of the *An. dirus* complex found in the *An. leucosphyrus* group, *An. dirus* and *An. baimaii*; two members of the *An. maculatus* group, *An. maculatus* and *An. sawadwongporni*; and two members of the *An. minimus* complex belonging to the *An. minimus* subgroup, *An. minimus* and *An. harrisoni*. Previous studies have successfully described the trophic behavior, biting activity, and seasonal abundance in the *An. maculatus* group and *An. minimus* subgroup [9,34], whereas no such studies have documented these activities for the two most important malaria vectors within the *An. leucosphyrus* group, *An. dirus*, *An. baimaii*.

*Anopheles baimaii* and *An. dirus,* are considered to be the primary malaria vectors in Thailand [2,35]. These two closely related species cannot be differentiated from each other by morphological characteristics alone [8,25]. Both are forest and forest-fringe mosquitoes that are considered highly anthropophilic [2,11]. The preferred breeding habitats of these two species are animal footprints, wheel-tracks and temporary ground pools in heavily shaded areas. In addition, larval habitats can be found in water jars, cut tree stumps, and root holes. *An. dirus* is the only species that is found throughout Thailand and often occurs in sympatry with *An. baimaii* in the western part of the country [36]. Both species are considered very anthropophilic in their blood feeding preference and demonstrate both exophagic and endophagic behaviors, and in some cases a generally greater tendency toward exophily [3,5,35]. However, interpretation of these early studies was hampered by species identification that was based on morphological characters, which was unreliable resulting in misidentification of species. This complicated the behavioral analysis of each sibling species within the complex [3,17]. Unlike these earlier studies, species of mosquitoes reported in this study were subjected to a multiplex AS-PCR, providing accurate species identification in order to describe with reliability the trophic behavior, seasonal abundance, and host preference of *An. dirus* and *An. baimaii* in Pu Tuey Village, Kanchanaburi Province. *An. dirus* demonstrated a strong zoophilic behavior. More mosquitoes were caught on cattle bait compared to human landing from both inside and outside houses (63.2% of *An. dirus*). Results of previous studies on biting activity and host preference of *An. dirus* complex in central Thailand demonstrated a delayed and more prolonged feeding peak that occurred between 2000 and 2400 h [13,14]. In southern Thailand, Scanlon and Sandhinand [12] reported the peak biting activity to occur between 2400 and 0300 h. Rosenberg *et al*. [15] observed a biting peak that occurred between 2200 and 0100 h, and Rattanarithikul *et al*. [16] reported a single early-evening biting peak between 2000 and 2200 h. In western Thailand, Sungvornyothin *et al*. [17] observed the biting activity between 2000 and 2100 h for both indoor, outdoor human landing collections and between 2000 and 2300 h in cattle baited collections. All of those biological and behavioral studies for *An. dirus* used only morphological characteristics for identification. This high degree of variability in biting patterns for populations found throughout Thailand might be the result of the mixed populations of species.

Our study described the biting behavior and blood feeding activities based on a clear and reliable identification of the sibling species of the *An. dirus* complex using PCR technology. The indoor biting activity of *An. dirus* recorded in this study demonstrated a peak between 1900 and 2000 h. Outdoor human landing collections identified a prominent peak between 2300 and 0100 h. On the other hand, cattle baited collections showed one clear peak in the early evening between 1900 and 2100 h, followed by a declining trend. *An. baimaii* was collected in a small proportion compared to *An. dirus* and showed an ambiguous pattern of feeding activity. Indoor numbers were too small to evaluate but outdoor activity peaked between 2400 and 0100 h. Cattle baited catches showed two clear peaks at both 1900–2100 h and at 2400–0100 h. Accurate identification of the species using multiplex PCR technology could explain the differences in feeding patterns of these sibling species complexes when compared to earlier published reports. Previous studies have shown that the morphological identification alone of the two sibling species of the *An. minimus* complex is not reliable and can result in nearly 40% misidentification of specimens [9,18].

The seasonal abundance of the *Anopheles* mosquitoes collected in this study appeared to be influenced by several factors. Adult densities were found to be positively associated with increased rainfall (July to August). This is clearly seen from the fact that the greatest numbers of adults were found during the wettest period of the year. Rainfall dependent abundance patterns have previously been reported for Thailand and Bangladesh [5,10,17,37]. This suggests that high rainfall provides adequate larval habitats for *An. dirus* that prefers temporary breeding habitats such as animal footprints, wheel tracks, and temporary ground pools common during the wet season. An inverse relationship with rainfall was documented for *An. minimus*[9] and *An. maculatus* in the same locality [34], wherein these two species have been shown to prefer breeding at the edges of slow-running streams [9,38]. Heavy rainfall would flush these habitats out resulting in reduced adult densities. In contrast, a negative association was found with a higher mean ambient temperature and relative humidity. Although, the number of malaria cases is low in Pu Teuy Village, the area remains at risk for increased transmission due to a high degree of human movement (parasite introduction) from highly malarious areas. Also, the current findings agree with previous work that demonstrated that efficient malaria vectors such as, *An. dirus*, *An. minimus* and *An. maculatus* are commonly found in greater abundance during the rainy periods of the year.

## Conclusions

Accurate identification of the species with the use of multiplex PCR technology could explain the differences in feeding patterns of these sibling species, *An. dirus* and *An. baimaii* in Pu Teuy Village as compared to previously published work from other localities in Thailand. The current findings confirm that these species have a higher propensity to feed outdoors compared to indoors as previously published. A better understanding of the members of the Dirus sibling species complex, *An. dirus* and *An. baimaii* behavior related to host preference and both temporal and spatial feeding activity will help facilitate the design and efficiency of malaria vector control operations in Thailand.

## Competing interests

The authors declare that they have no competing interests.

## Authors’ contributions

All the authors have contributed significantly to this study. TC and JPG assisted with study design. CT, RT and TC did the laboratory and field data collection. SM and WJ carry out the molecular genetic studies. AP performed statistical analysis. All authors contributed to the production of the manuscript and revision, with TC as final guarantor. All authors read and approved the final version of the manuscript.
